# The lived experiences and social support needs of first-time mothers at health care facilities in the City of Tshwane, South Africa

**DOI:** 10.4102/curationis.v40i1.1680

**Published:** 2017-09-22

**Authors:** Mmajapi E.T. Masala-Chokwe, Tendani S. Ramukumba

**Affiliations:** 1 Adelaide Tambo School of Nursing Science, Tshwane University of Technology, South Africa

## Abstract

**Background:**

Social support refers to the assistance people receive from others, and it is divided into four types of support. Given the increasing mortality and morbidity rates of mothers and neonates postpartum, this study intended to determine whether the social support needs of the first-time mothers were met after early discharge from health care facilities.

**Objectives:**

The objective of the study was to explore the lived experiences and social support needs of the first-time mothers after an early discharge from health care facilities in the City of Tshwane, Gauteng.

**Method:**

A qualitative explorative study was conducted to explore the lived experiences and social support needs of the first-time mothers. The population were first-time mothers who had a vaginal delivery and were discharged within 6–12 hours of delivery from health care facilities. Purposive sampling was performed and 14 semi-structured interviews were conducted, with those mothers who came for the prescribed three postnatal check-ups at the three health care facilities identified according to maternity services provided. Saturation of data for the three health care facilities was reached at the 14th interview. Data analysis was performed using the hermeneutic interpretive approach.

**Results:**

Almost all participants had completed grades 11 or 12, but most were unemployed. The needs identified included the need for social support, lack of confidence, knowledge and skill to care for themselves and their newborn babies after early discharge.

**Conclusion:**

There is need to identify alternative types of social support for the first-time mothers, to ensure a normal adjustment to motherhood.

## Introduction

The postpartum period is defined as the first six weeks after birth, and a critical period of health and survival of the mother and of the newborn baby (World Health Organization [WHO] 2014). Social support refers to the assistance people receive from others, and it is divided into emotional, instrumental, informational and appraisal support (House 1981). Furthermore, social support has a buffering and health benefit effect during the stressful time of adjusting to a new situation, such as being a first-time mother (House, Umberson & Landis 1988). Although scientific evidence does not indicate an increase in maternal and neonatal mortality and morbidity after early discharge, concerns about possible complications exist among health care providers concerning first-time mothers (Bravo, Uribe & Contreras 2011). The WHO (2013) has programmes designed for reproductive and child health care provision; however, postpartum care programmes are among the weakest of all programmes in Africa. According to the Guidelines for Maternity Care in South Africa (2015), pertaining to postnatal care, the mother and the baby should visit the clinic once at three and six days postpartum and the last visit should be at six weeks postpartum (Department of Health 2015). The purpose of the study is to report on the lived experiences and social support needs of first-time mothers after early discharge from selected health care facilities in the City of Tshwane, Gauteng.

## Problem statement

Becoming a mother for the first time is a life-changing experience and major life adjustments have to be made (Deave, Johnson & Ingram 2008). The characteristic present-day nuclear or single-parent family structure results in limited support structures for the first-time mothers after discharge, compared to the potential support that a new mother had received in an extended family structure (Wiemers et al. 2016). The situation might be aggravated by the current practice of early discharge of first-time mothers from health care facilities before they could master the care required by their babies and adjust to their mothering role (Kurth et al. 2016). Given the above facts, the researchers explored the experiences of the first-time mothers and their need for social support in order to determine whether the current early discharge practice meets the support needs of first-time mothers.

The current practice of early discharge postpartum at 6–12 hours after a normal vaginal delivery minimises the opportunity for first-time mothers to master the skills and knowledge of caring for themselves and their newborn babies when at home. Anecdotal evidence shows that mothers expressed lack of confidence when caring for themselves and their babies.

## Objectives

The objective of the study was to explore and describe the lived experiences and the social support needs of the first-time mothers after early discharge from health care facilities in the City of Tshwane, Gauteng, from the immediate postpartum period to 6 weeks postnatally.

## Background and trends

Challenges that contemporary mothers face because of changes in family structure, maternal role development and early post-partum discharge from health care facilities put added demands on these mothers’ emotional and physical adjustments during the postnatal period (Emmanuel et al. 2011). Limited human capacity in maternity care centres, the increasing pressure on hospital beds and limited physical space to care for mothers and their newborn babies contribute to early discharge of postnatal mothers and their babies (Emmanuel et al. 2011; Foster et al. 2008). The policy and practice of hospitalisation duration decreased to 6–12 hours in most countries after normal vaginal births and 48–72 hours after low-risk caesarean births (Brown et al. 2008).

Early discharge of new mothers and their babies from maternity units and health care facilities further creates a lack of professional support during the first 6 weeks postpartum (Barimani et al. 2014). Benning and Paladine (2005) mentioned the key potential danger signs to be detected and treated timely during postpartum care of the mother and the baby from 6 hours to 7 days postpartum. Some of the potential danger signs identified within six days postpartum for the mother include blood loss, pain and painful breasts, while potential danger signs for the baby include abnormal breathing, high temperature, feeding problems and infection of the umbilical cord. First-time mothers might lack confidence in caring for themselves and their newborn babies postpartum and it is not known who the first-time mothers consult when they encounter health problems (WHO 2013). This calls for integrated interventions.

The WHO (2013) suggests 12 recommendations as guidelines for the care and management of the mothers and their newborn babies, one of which is based on psychosocial support of the mother by a trained person. Warren et al. (2008) stated that every year about 125 000 women and 870 000 newborns die in Africa during the first week after birth. In addition, the WHO (2013) states that post-delivery programmes are rated at the lowest priority along the continuum of care.

In response to the increasing number of postpartum deaths and complications, the WHO (2013) prescribes the timing, contact and content of postpartum care to be provided to postpartum women especially in low-resource communities. Warren et al. (2008) stated that half of all the postpartum maternal deaths occur during the first week after birth, and many of these deaths may occur during the first 24 hours. Hence, in most African countries, including South Africa, women and their babies should attend health care facilities at 3 days, 7 days and 6 weeks after an early discharge. There appears to be a dire need to identify factors contributing to maternal and neonatal mortality and morbidity after early postpartum discharge. If complications are identified and treated early, the maternal and neonatal mortality and morbidity rates might decline in South Africa.

The Strategic Plan for Maternal, Newborn, Child and Women’s Health and Nutrition in South Africa 2012–2016 (Department of Health 2010) stipulates that the priority interventions for reducing maternal and child mortality rates are to make sure that every woman receives a comprehensive primary health care (PHC) package. This package specifies that postpartum care must be home-based to ensure that the mother and baby are screened for possible risks in the environment where they live. The objectives of the home-based care visit include (Department of Health 2010) newborn care and promoting and supporting exclusive breastfeeding. According to Barimani et al. (2014) little is known about where mothers turn for help with the challenges related to the postpartum period. Hence, the need to explore the lived experiences of social support needs of the first-time mothers after an early discharge from health care facilities in the City of Tshwane, Gauteng.

### Definition of terms

Early discharge: It is the practice of discharging mothers and their babies within 6–12 hours post-delivery of a normal vaginal delivery and within 72 hours of a low-risk caesarean section.

Low-risk caesarean section: This is a caesarean section which was performed for foetal reasons or indications, for example foetal distress, and the caesarean section was performed on a healthy pregnant woman who had no chronic medical conditions.

Health care facilities**:** In this study, these are the PHC centres which provide level one, two and three health care to mothers and babies. These are PHC facilities which are the communities’ point of access to the health system.

First-time mothers: Women who are becoming mothers for the first time; women who had given birth to their first newborn babies.

Postpartum period: Sometimes referred to as postnatal period is the period after the birth of a baby and commences from the first hour after delivery of a baby and ends at 6 weeks thereafter.

Postpartum care: Sometimes referred to as postnatal care or post-delivery care includes the assessments and care provided to mothers and babies after birth, and end at 6 weeks thereafter.

Immediate postpartum care**:** This refers to the care provided to the mother and the baby within the first 24 hours after the baby’s birth.

Social support: This is defined as the hopefulness, caring and reliance that the postpartum first-time mothers expect from midwives, families and communities up to 6 weeks after their discharge from health care facilities.

### Contribution to the field

The findings of the study added information about some of the possible effects of early discharge on the first-time mothers. The needs for social support during the postpartum were highlighted by the study. These findings also indicated a need for an alternative measure such as a social support programme for the first-time mothers that could provide social support in the absence of an extended family structure. There is a need to relook at the practice of early discharge, especially for the first-time mothers and to search for alternative forms of social support.

## Research method and design

### Design

A qualitative, descriptive and exploratory design was adopted. The motivation for using an exploratory design was to understand the meaning that the participants attributed to their everyday lives about early discharge and their postpartum social support needs (Creswell 2007:36). In the current study, the researchers explored the lived experiences and social support needs of the first-time mothers from early discharge up to their postnatal clinic visits at 6 weeks after their babies’ births.

### Context of the study

This study was conducted at three health care facilities in the City of Tshwane, a metropolitan municipality in Gauteng Province, with a population of about 2 million people. The health care facilities comprise academic hospitals which are level two and three, while level one are PHC facilities. The PHC facilities, providing maternal and child health care service, fall within level one. Out of the three health care facilities identified for the study, two were open only during the day for 5 days a week. The remaining health care facility was open for 24 hours and operated as a midwifery obstetric unit (MOU), a PHC facility and was open for 7 days a week.

The area surrounding the participating health care facilities comprised mostly Setswana-speaking people residing with six other languages and cultural groups namely the BaPedi, Basotho, BaVhenda, Zulus, Xhosas and Tsongas. The justification for using the three health care facilities was that they offered maternal and child health services to most postpartum mothers and their babies residing in the area, during their postnatal clinic visits for a check-up. According to the interim report of the National Committee for Confidential Enquiry into Maternal Deaths of 2011–2013, the average childbearing age of mothers in South Africa ranged from less than 20 years to more than 45 years of age (Department of Health 2014).

### Population and sampling

The accessible population comprised the postpartum first-time mothers who were discharged within 6–12 hours from health care facilities in the City of Tshwane. The first-time mothers were asked about their experiences of early discharge, social support needs and taking care of themselves and their babies at home. A non-probability purposive sampling method was used to recruit participants at the three health care facilities in the chosen community. The three health care facilities were chosen as they provided postnatal care services to women who had given birth at those facilities while some had given birth at a level-two hospital and some at the tertiary hospital. All the postpartum first-time mothers who attended one of the three health care facilities, and were residing in these health care facilities’ surrounding areas, were invited to participate in the study during their postnatal visits at the facility for a check-up.

The inclusion criteria were postpartum first-time mothers who visited one of the three participating health care facilities for their postnatal check-ups, resided in the chosen communities, whose babies were born normally or through low-risk caesarean sections, were discharged within 6–12 hours after giving birth, and could communicate either in English or Setswana.

The exclusion criteria were mothers who were discharged after 12 hours of delivery, were more than 6 weeks postpartum, who were not first-time mothers, did not reside within the chosen community or had a complicated caesarean section.

### Data collection procedure

Arrangements were made with the managers of the three identified health care facilities to visit these facilities once a week to gather dense data. On the day of data collection, the first author approached all postpartum first-time mothers who were attending the PHC facility, who complied with the inclusion criteria, invited them to participate in the study, and all indicated their availability and willingness to participate. The first author explained the type of research and supplied leaflets with information and consent forms. The women who were willing to be interviewed were requested to sign consent forms.

An interview schedule consisting of two sections was formulated. Section A gathered demographic data required to describe the sample. This included aspects of age, level of education, occupation and level of income. Section B comprised open-ended questions and a few probes which the researcher followed during the interview. The interview schedule was translated into Setswana to facilitate interviewing those who could not communicate in English.

The interview schedule was pre-tested by conducting interviews with two postpartum first-time mothers at the health care facility who were not included in the study. The biographical information section required re-numbering and two questions had to be rephrased after the data obtained during the pre-test had been analysed.

At the health care facility, after introducing herself and the field worker to all postpartum women and explaining the reason for the visit, the first author conducted individual interviews in a private room. Permission to interview the participants and the use of the audio tape recorder was requested from them and they signed a consent form to indicate their willingness to participate. The use of the audio tape recorder was to ensure that the data transcribed were a true reflection of what was shared by the participants.

Interviews were conducted by using an interview schedule (Greeff 2005). The interviewer asked the central questions, and probed while the field worker managed the audio recording and took field notes. The moderator made sure that there were no unnecessary disturbances during the interviews and later played the role of the intercoder. The central questions were followed by probing, paraphrasing and reflecting in order to clarify the meanings of the data collected. Data were collected once a week, where three to five first-time mothers were interviewed.

During the interviews, the participants unfolded their experiences of being discharged from health care facilities within 6–12 hours after their babies’ birth and their social support needs. For this purpose, open-ended questions were asked, which read: ‘Please tell me about your experience of being discharged early from the health care facility, your social support needs and your experience of taking care of yourself and the newborn baby at home, and tell me how you recognised whether your baby was well or not.’ (Authors’ own phrase)

The questions were followed by probes, reflections and paraphrasing. The interview sessions lasted approximately 45 minutes.

Data saturation was reached at the 14th interview as no new information emerged from subsequent interviews.

### Data analysis

A hermeneutic interpretive approach was used to analyse the data. Speziale and Carpenter (2007) identified the hermeneutic interpretive approach of analysing the data in qualitative research as comprising three-step processes namely naïve reading, structural analysis and interpretation of the whole. Data analysis commenced after the first three interviews had been conducted. The data analysis process commenced with the verbatim transcribing of the information from audio tapes. During this process, the researcher immersed herself in data referred to as ‘dwelling within the data’ (Burns & Grove 2011). The transcriptions were read and re-read to identify different subcategories, then categories. Similar categories were later classified into themes.

The biographical data were analysed quantitatively by using descriptive statistics indicating the frequency and percentage distributions of variables (Brink, Van Der Walt & Van Rensburg 2012). The independent coder was used to limit bias during the data analysis (Lincoln & Guba 1985:Chapter 11). A consensus discussion was held between the researcher and independent coder to determine intercoder reliability.

### Ethical consideration

The study received approval from the University’s Ethics Committee (FCRE 2011/03/004), the Tshwane Research Committee and the managers of the three health care facilities where the study was conducted. Each participant signed a consent form before being interviewed. Respect of persons, beneficence, confidentiality, anonymity and autonomy were adhered to (Brink, Van Der Walt & Van Rensburg 2012).

## Results

The results are presented as the demographic profile of the participants and the themes which emerged during data analysis.

### Demographic profile of the first-time mothers

The demographic profile of the participants is shown in Table 1.

**TABLE 1:** Demographic characteristics of the participants (*N* = 14).

Characteristics	*n*	%
**Age**		
18–20	8	57.1
21–30	2	14.3
31–40	4	28.6
> 41	0	-
**Marital status**		
Married	3	21.4
Single	11	78.6
Widowed	0	-
Divorced	0	-
**Highest education**		
Tertiary	1	7.1
Grades 11–12	12	85.7
Grades 8–10	1	7.1
Up to Grade 7	0	-
Never attended school	0	-
**Occupation**		
Formal worker	6	42.9
Unemployed	7	50.0
Self-employed	1	7.1
**Monthly income**		
> R5000	2	14.5
R4999–R3001	2	14.5
R3000–R2500	1	7.1
R2499–R1100	1	7.1
< R1000	1	7.1
None	7	50.0
**Methods of baby feeding**		
Breastfeeding	13	92.9
Bottle feeding	0	-
Mixed feeding	1	7.1

**Total**	**14**	**100**

*Source:* Authors’ own work

As much as 57.1% (*n* = 8) of the first-time mothers were younger than 21 years of age while 42.9% (*n* = 6) were between the ages of 21 and 40. Most participants 78.6% (*n* = 11) were single (unmarried) mothers. Out of the 14 participants, 86.7% (*n* = 12) reportedly had passed Grades 11 or 12, one had tertiary education and one had a Grade 8–10. Half of the participants (50.0%; *n* = 7) were unemployed while 42.9% (*n* = 6) had formal work and one was self-employed selling vegetables. Almost all participants (92.9%; *n* = 13) breastfed their babies, and one (7.1%) gave both breast milk and porridge to the baby. None of the participants gave their babies formula feeds.

### The first-time mothers’ lived experiences and social support needs

Table 2 outlines the main themes that emerged from the data analysis, namely the need for support, knowledge and skills related to baby care and cultural beliefs. Categories and subcategories were identified as summarised in Table 2. Direct quotes are used to support the findings. In order to ensure anonymity, the participants are named using the alphabet for example Participant M means participant number 13 (Alphabet) and Participant D is participant number 4.

**TABLE 2:** Lived experiences and social support needs of first-time postpartum mothers.

Themes	Categories	Subcategories
The need for support	Lack of confidence in mothering	Feelings of doubt, fear and anxiety
		Desperation to become a ‘picture-perfect mother’
Knowledge and skills deficit related to baby care	Baby care	Bathing and holding the baby General well-being of the baby Umbilical cord care
	Breastfeeding as a challenge	Concerns about breastfeeding
Cultural belief	Cultural beliefs and myths	The relationship between the umbilical cord, passing stools and urination

*Source:* Authors’ own work

#### The need for social support

The postpartum first-time mothers repeatedly expressed that they lacked confidence and needed social support. They had doubts about how they would care for themselves and the babies. These experiences were discussed under lack of confidence in mothering as expressed by feelings of doubt, anxiety and desperation to be a perfect mother.

Lack of confidence in mothering was revealed by participants who experienced uncertainty and lacked confidence to care for their babies which they related to their early discharge from the health facilities. Some women said that they felt guilty to have a meal when their babies sucked nothing from their empty breasts during the immediate postpartum period. In one incident the baby was separated for 24 hours from the mother because of ill health and was observed in the neonatal intensive care unit. The mother and baby were united for the first time when they were discharged from hospital. No assessment was performed on the woman to determine whether her milk supply would be sufficient, as the baby had never been breastfed prior to discharge. Feelings of doubt and anxiety evoked further strong emotions such as frustration and confusion. Most of the first-time mothers stated that they were holding a newborn baby for the first time in their entire lives, and expressed the lack of confidence as follows:

‘I worry a lot about what type of a mother I will be. I sometimes stress, feel down and, depressed, as I’m worried about how to bring up the baby.’ (Participant M, 18 years, Grade 11)

‘My sister encourages me to continue. She says it will become better with time. Because I love my baby too much, I believe her.’ (Participant G, 18 years, Grade 12)

‘My family helped me and taught me how to hold the baby.’ (Participant K, 20 years, Grade 12)

‘My partner asks things about the baby, why is the baby changing colour, why the cord is not falling off, and I don’t have answers for him. I am blank … I know nothing about the baby.’ (Participant C, 18 years, Grade 9)

The participants indicated that they were concerned about the type of mothers they would be and most of them expressed their wishes to be good and perfect mothers. Because of these aspirations some participants could not sleep as they were unsure about the safety of their babies while they slept. Some mothers reportedly spent most of the night checking that their babies were breathing while some were wary of leaving their babies with wet or soiled nappies at night, requiring them to stay awake at night. All these expressions indicated that the postnatal women were overly concerned to keep their babies safe. The participants wished to be the ‘perfect mothers’ or else they might be blamed later if something went wrong with themselves or their babies. As a result, the participants continued to deprive themselves of sleep and rest required for their postpartum recovery. This is what participants said:

‘What if something goes wrong, affecting me or my baby? What if my baby stops breathing, what will I do?’ (Participant D, 32 years, Grade 12)

‘I check her every time, throughout the night. I am worried about the safety of me, and I worry a lot about this.’ (Participant E, 30 years, Grade 11)

‘The baby slept here and there. But I kept awake most of the time to make sure that she is well, … is breathing, is warm and nappy is dry.’ (Participant K, 20 years, Grade 12)

#### Knowledge and skills deficit related to baby care

**Baby care**: The lack of knowledge and skill related to bathing and holding the baby and how to determine when the baby is not well. Some of the participants stated that they thought bathing the baby is like taking a bath: they soaped the washing rag and bathed the baby like they would bathe themselves. Some viewed holding the baby either on the breast or just comforting the baby to be posing challenges. Some participants were too scared to hold the baby because they did not know how to hold the newborn baby, fearing that they might ‘drop the baby’, or ‘break the baby’ or ‘hurt the baby while holding him’. Some of the participants said:

‘I was afraid to hold the baby; I thought the baby will break. I did not know how to hold the baby.’ (Participant A, 19 years, Grade 11)

‘I am scared that I might drop the baby. It is too slippery.’ (Participant G, 18 years, Grade 12)

‘How does one test if the temperature is alright for the baby? Why don’t I know all these?’ (Participant G, 18 years, Grade 12)

‘If she does not wake up, after how long, must I remind her by waking her up for feeding?’ (Participant I, 19 years, Grade 12)

It dawned on most participants during the interviews that they would be unable to identify a baby who is unwell. However, a few could explain some of the signs which could indicate that the baby was sick if the baby refused to feed, ‘felt hot to touch’ or passed loose stools.

When asked about the signs of a baby who is not well, some participants stated:

‘I don’t know. I have never thought about that, now I realise I don’t know.’ (Participant E, 30 years, Grade 11)

‘Never thought about this … [*smiling*]. I’m … just a guess … not eating, her face not giving the usual look, crying a lot.’ (Participant D, 32 years, Grade 12)

‘If the baby does not do what I am used to, or if he wakes up earlier than usual, or when he is not feeding well.’ (Participant J, 22 years, Grade 12)

Umbilical cord care was a problem. Some first-time mothers were afraid of touching or cleaning the umbilical cord and mentioned that they hated the crying of their babies when the umbilical cord was cleaned or touched. They believed that the baby felt pain when the cord was touched. Some participants mentioned that they walked away as soon as a support person started cleaning the umbilical cord, stating:

‘The way the baby cries and screams when the cord is cleaned, it scares me.’ (Participant E, 30 years, Grade 11)

‘I literally go out when my sister attends and cleans the umbilical cord.I did not know that the cord must be kept exposed at all times to encourage drying off.’ (Participant A, 19 years, Grade 11)

‘I was afraid of the stump of the cord.’ (Participant K, 20 years, Grade 12)

**Breastfeeding was a challenge:** Most participants viewed breastfeeding as a challenge. Some participants mentioned that although they were able to breastfeed the babies, they did not know when the baby was satisfied. As a result, some articulated worries that the baby would continue to breastfeed without stopping, hence they had to ‘stop’ the baby from breastfeeding to control the amount taken during breastfeeding.

Some participants mentioned that it always seemed easy when they read books about breastfeeding, but it was difficult when it came to applying what they had ‘learned’ in real life. Most of the first-time mothers complained about painful nipples, especially when breastfeeding for the first time. They mentioned that the pain on the breast felt like the ‘baby was biting the nipple’. Some of the participants alluded:

‘We have been taught how to breastfeed and hold the breast for the baby at the clinic, I thought this will be easy. The challenge is when coming to the practical part of it, reality strikes … and I realised I don’t know how.’ (Participant F, 21 years, Grade 11)

‘My body was painful. On the first day, no milk came out and the baby was crying. It only came out from the second day, and the baby is no more crying a lot.’ (Participant E, 30 years, Grade 11)

‘The baby was continuously sucking and I don’t know for how long the baby should continue. What will show me that the baby is full? I don’t know.’ (Participant F, 21 years, Grade 11)

#### Cultural belief and myths

Cultural practices and beliefs related to the umbilical cord, breastfeeding and passing of stools. They stated that they were worried that the babies did not pass urine, and the babies were crying when passing stools.

Some participants believed that the umbilical cord was related to other systems of the baby’s body. For instance, the baby might not pass urine or might have difficulty passing stools as long as the umbilical cord had not fallen off. Some participants mentioned that their babies cried when passing stools and some stated that their babies did not pass stools every day, and all these aspects were related to the umbilical cord which had not fallen off. The participants mentioned:

‘Because they told me he will not pass stools normally until the cord falls off. They told me I must not worry; he will not pass stools every day until the cord falls of. After the cord has fallen off, he will pass stools normally, that is every day.’ (Participant E, 30 years, Grade 11)

‘As long as the umbilical cord is on I know my baby will not pass urine.’ (Participant A, 19 years, Grade 11)

## Trustworthiness, validity and reliability

Credibility and transferability were enhanced by the fact that the first author had more than 20 years’ experience as a midwife while the second author is a recognised researcher. Consistency was maintained by ensuring that the first author conducted all the interviews using the same interview schedule.

### Validity

Validity was ensured by the fact that the study’s proposal was approved by the Ethics Committee of the university. The scientific processes of sampling, data collection and analysis were followed and all the steps were supervised by the two researchers who were the supervisors of the study. The first author was a doctoral candidate, supervised by researchers with vast experience in community nursing and research. The environment and context of the study were described. Content validation of the tool was attended to by using relevant literature.

### Reliability

Reliability is the extent to which an experiment, test or any measuring procedure yields the same result on repeated trials (Burns & Grove 2011). In this study, pretesting of the instrument was performed to ensure that the instrument measured what intended to measure. Analysis of data was performed by the researcher and the independent coder separately from each another. Later, the results were compared, and consensus was reached.

## Discussion

### Outline of the results

The women in the current study identified the need for social support, as they lacked knowledge and practical skills to provide baby care. Cultural practices and expectations were identified, which could guide the women’s care and social support needs. The current South African system of early postnatal discharge does not meet the needs of the first-time mothers, resulting in high anxiety levels.

First-time mothers expressed a lack of confidence characterised by feelings of doubt, anxiety and fears while caring for themselves and their newborn babies in the current study. These first-time mothers did not know about which signs and symptoms could indicate that the baby might be sick. Danbjorg and Clemensen (2014) reported that because of the practice of early postnatal discharge, Danish parents lacked follow-up support from the health professionals about potential questions, and hence they developed insecurities. The current study’s findings revealed that postpartum first-time mothers suffered from sleep deprivation as they tried to be ‘perfect mothers’ to their babies. They spent much time during the nights stressing about their babies’ well-being; by so doing they delayed their postnatal recovery which might impact on the development of the maternal role. An Australian study revealed that maternal role development could be compromised by maternal distress (Emmanuel et al. 2011) and a lack of sleep. Sleeplessness could aggravate maternal distress and increase anxiety levels leading to postnatal depression if the situation was not addressed. The effects of maternal confidence and competence on postpartum mothers’ parenting stress, maternal competence and confidence were important factors for minimising stress, doubts and anxiety (Liu et al. 2012).

In the current study, some mothers believed that a baby cried while passing a stool because the umbilical cord was still on. Although there is no direct danger about such beliefs, these myths should be clarified by a health professional. Most cultures have rituals connected to the postpartum period. There has been the practice of confining the postpartum mother to the house for 40 days in Norway as the woman was considered to be unclean (Eberhard et al. 2010).

### Practical implications

The first-time mothers in the current study experienced a lack of knowledge and skills to care for themselves and their babies. The first-time mothers indicated their lack of basic skills and knowledge on how to hold the baby during breastfeeding and how to bathe their babies. The first-time mothers in the current study only realised when they arrived at home that they lacked information about simple tasks like how to recognise that the baby was not well and how to determine whether the baby was satisfied after a feed. Umbilical cord care and breastfeeding were viewed as being challenges. Although they were taught about breastfeeding during the antenatal period, the reality of breastfeeding their own babies had its own difficulties. This was also shown in a study about postnatal depression among first-time mothers, indicating that they had to learn new skills and roles which would help them to adjust to the new mothering role (Leahy-Warren, Mccarthy & Corcoran 2011). The expression of a lack of knowledge and skills to care for their babies indicates a need for support and empowerment from the family, care workers or the community in the absence of a health professional (Leahy-Warren et al. 2011). Ellberg and Lindh’s (2010) Swedish study revealed that both first-time and multiparous mothers needed support to adjust to their individual needs and regarded the support and help they received from the health professionals as being insufficient (Ellberg & Lindh 2010).

## Limitations of the study

Only first-time mothers were interviewed. Perhaps the findings might have been different if all mothers within the 6-week postpartum were interviewed. The findings cannot be generalised to all first-time mothers in other areas in Tshwane unless the study has been repeated in other areas.

## Recommendations

There is a need to reconsider the practice of early discharge as the practice does not meet the social support needs of first-time mothers. Alternative social support structures should be identified within the community to cater for the first-time mothers during the postpartum period. There is a need to develop social support groups for first-time mothers in the community. In addition, the available community health care workers who are performing door-to-door follow-up visits for patients with chronic medical conditions within the community could be trained to provide social support to the first-time mothers.

## Conclusion

The strength of this study is that it has provided an in-depth exploration of the experiences and social support needs of 14 first-time mothers in the City of Tshwane which has provided important insights into the current provision of post partum care.
